# Adhesion and colonization of mesenchymal stem cells on polylactide or PLCL fibers dedicated for tissue engineering

**DOI:** 10.1186/1753-6561-7-S6-P53

**Published:** 2013-12-04

**Authors:** Frédérique Balandras, Caroline Ferrari, Eric Olmos, Mukesh Gupta, Cécile Nouvel, Jérôme Babin, Jean-Luc Six, Nguyen Tran, Isabelle Chevalot, Emmanuel Guedon, Annie Marc

**Affiliations:** 1 CNRS, Laboratoire Réactions et Génie des Procédés, UMR 7274, Université de Lorraine-ENSAIA, 2 avenue de la forêt de Haye, TSA 40602, F-54518 Vandoeuvre-lès-Nancy Cedex, France; 2 CNRS, Laboratoire de Chimie Physique Macromoléculaire, FRE 3564, Université de Lorraine-ENSIC, 1 rue Grandville, 54000 Nancy Cedex, France; 3 École de Chirurgie, Faculté de Médecine, Université de Lorraine, F-54500 -Vandœuvre-lès-Nancy, France

## Background

Tissue engineering covers a broad range of applications dedicated to the repair or the replacement of part or whole tissue such as blood vessels, bones, cartilages, ligaments, etc [1]. Practically, a bio substitute, made with cells cultivated on scaffold, is needed. Mesenchymal stem cells (MSC) are generally the most suitable cells for such application since they are self-renewable with a great potential for differentiation and immuno suppression [2]. However, materials used for bio functional scaffold synthesis have to meet several criteria, such as biocompatibility and biodegradability. Thus, the aim of the study was to screen several biopolymers differing in their composition for their capability to promote adhesion and growth of MSC.

## Materials and methods

Porcine MSC were cultivated in α-MEM supplemented with 10% serum and FGF2. For cell adhesion experiments, 6(co)polymers (Table 1) were synthesized and tested.

**Table 1:** Composition of co-polymers used in this study

Commercial PLCL	70% L-LA	30% CL
MKG58	70% D, L-LA	30% CL
MKG64	-	100% CL
MKG70	50%L-LA	50% CL
MKG71	100%D,L-LA	-
MKG74	100%L-LA	-

LA: lactic acid; CL: ε-caprolactone

Fibres of polymers were electrospun on 4 cm^2 ^cover glasses. Briefly, the polymer solutions are introduced into a syringe with various flow rates and an electrical field is applied, resulting in the formation of a polymer jet on cover glasses or on any surfaces. Then, cover glasses were put onto 6 wells plate before to be seeded with MSC. Then, cell adhesion and colonization of polymer fibres were monitored by microscopy and counted using Guava Viacount assay after trypsine treatment as already described [3].

## Results

**Figure 1:**
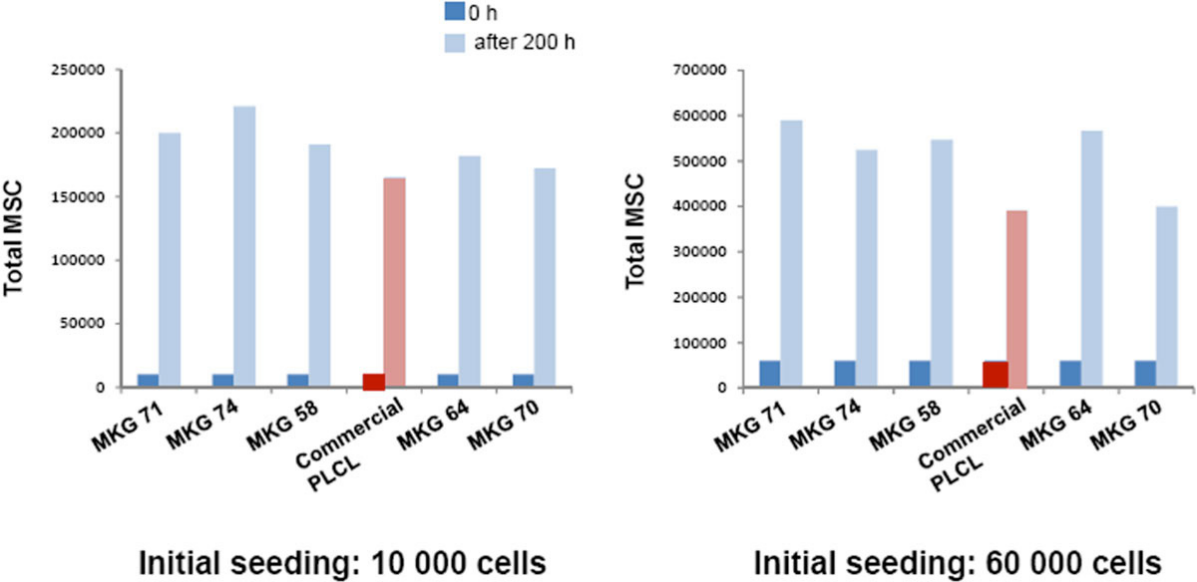
**Quantitative evaluation of MSC growth on poly lactide/caprolactone polymers**. Two initial MSC seeding, 10 000 and 60 000 cells, were carried out. The red stars indicate a significant increase in final cell number compared to the control (commercial PLCL).

With the aim of studying and identifying new materials dedicated to scaffold manufacturing for tissue ingineering, MSC were cultivated on various (co) polymers. These polymers, made with lactic acid (L and/or D) and/or caprolactone (blue bars; MKG 58, 64, 70, 71 and 74) in comparison with a commercial PLCL (red bars), were electrospun on cover glasses in order to functionalize them. Then MSC were cultivated on theses functionalized cover glasses at two initial cell seeding (10 000 and 60 000 cells) during 200 hours (Figure 1).

Whatever the polymer used and the initial cell seeding, cells were able to adhere and to colonize fibres. A cell multiplication factor ranging from 6.5 to 22 was measured after 200 hours of culture depending on the polymer composition and the initial seeding. However, compared to the commercial PLCL, the total cell number was strongly increased with MKG 71 (21 and 50%), MKG 74 (34 and 34%) and MKG 58 (15 and 40%) whereas a moderate growth was observed with MKG 64 (9 and 30%) at an initial seeding of 10 000 and 60 000 cells respectively. MKG 70 did not improve the cell growth compared to the commercial polymer (> 5% for both seeding).

## Conclusion

In this study, porcine MSC were cultivated on various (co)polymers made with lactic acid (L and/or D) and/or caprolactone. Our results demonstrated that composition of these (co)polymers strongly influences MSC growth and colonization. Indeed, polymers such as MKG 58, 71 and 74 appeared to promote MSC growth contrary to other polymers tested, i;e MKG 64 and MKG 70, compared to the commercial one. Therefore, MKG 58, 71 and 74 could be favoured for further scaffold synthesis.
